# BETA prime: a first-in-man phase 1 study of AdAPT-001, an armed oncolytic adenovirus for solid tumors

**DOI:** 10.1038/s41417-023-00720-0

**Published:** 2023-12-25

**Authors:** Anthony P. Conley, Christina L. Roland, Alberto Bessudo, Brian R. Gastman, Victoria M. Villaflor, Christopher Larson, Tony R. Reid, Scott Caroen, Bryan Oronsky, Meaghan Stirn, Jeannie Williams, Erica Burbano, Angelique Coyle, Minal A. Barve, Naveed Wagle, Nacer Abrouk, Santosh Kesari

**Affiliations:** 1 Department of Sarcoma Medical Oncology, The University of Texas MD Anderson Cancer Center, Houston, TX 77030 USA; 2 California Cancer Associates for Research & Excellence, San Diego, CA 92127 USA; 3 Department of Dermatology & Plastic Surgery, Cleveland Clinic, Cleveland, OH 44195 USA; 4 Department of Medical Oncology & Therapeutics Research, City of Hope, Duarte, CA 91010 USA; 5 EpicentRx, Inc., La Jolla, CA 92037 USA; 6 Mary Crowley Cancer Research, Dallas, TX 75230 USA; 7 Pacific Neuroscience Institute and Saint John’s Cancer Institute at Providence Saint John’s Health Center, Santa Monica, CA 90404 USA; 8 Clinical Trials Innovations, Mountain View, CA 94040 USA

**Keywords:** Sarcoma, Drug development

## Abstract

AdAPT-001 is an oncolytic adenovirus (OAV) with a transforming growth factor beta (TGF-ß) trap, which neutralizes the immunosuppressive and profibrotic cytokine, TGF-ß. The aim or purpose of this phase 1 study was to assess the safety and tolerability and, secondarily, the efficacy of AdAPT-001 after single intratumoral injection (IT) (Part 1) and multidose IT injection (Part 2) in patients with superficially accessible, advanced refractory solid tumors. Part 1 enrolled 9 patients with a 3 + 3 single dose-escalation safety run-in involving 2.5 × 10^11^, 5.0 × 10^11^, 1.0 × 10^12^ viral particles (vps). No dose-limiting toxicities or treatment-related serious adverse events (SAEs) were seen. In Part 2, a dose-expansion phase, 19 patients received AdAPT-001 at 1.0 × 10^12^ vps until disease progression according to Response Evaluation Criteria in Solid Tumors or RECIST 1.1. The overall responses to treatment included confirmed partial responses (3), durable stable disease ≥ 6 months (5), and progressive disease (13). AdAPT-001 is well tolerated. Evidence of an anti-tumor effect was seen in both injected and uninjected lesions. The recommended Phase 2 dose was 1.0 × 10^12^ vp administered by intratumoral injection once every 2 weeks. Combination of AdAPT-001 with a checkpoint inhibition is enrolling.

## Introduction

Oncolytic virus (OV) therapy is a potentially useful treatment strategy especially in the wake of the success of checkpoint inhibitors to generate de novo or boost pre-existing native immune responses. Firstly, OVs selectively infect and lyse cancer cells, taking advantage of the tolerogenic mechanisms, which operate in tumors, and therefore act as an in-situ cancer vaccine via the release of tumor-associated and tumor-specific antigens and danger signals [1]. Secondly, they have the potential to beneficially remodel the tumor microenvironment from cold or non-inflamed to hot or T-cell inflamed for more effective anticancer immunity through a domino effect of cell lysis, transgene expression, antigen presentation, and adaptive immune activation [2]. Thirdly, given their minimal, nonoverlapping toxicities, they may combine well with immune checkpoint inhibitors whose activity depends on the presence of tumor-specific T cells [3].

Adenovirus type 5 (Ad5) is a highly lytic virus, agent of the common cold, whose progeny virions spread to neighboring cells for successive rounds of infection and lysis. For over two decades, attenuated oncolytic Ad5 vectors have accumulated an extensive safety record in clinical trials. However, despite high hopes, only one oncolytic adenovirus (OAV) has ever been approved; Oncorine, formerly known as Onyx-015, was licensed by the Chinese FDA in 2005 for the treatment of late-stage refractory nasopharyngeal cancer combined with cisplatin and/or 5-fluorouracil (5-FU) [4].

AdAPT-001 was designed with the following properties: (1) minimal modification. A small 50 base pair deletion in the E1A promoter region renders AdAPT-001 non-lytic in normal cells but lytic in cancer cells [5, 6]. (2) a fast replication phenotype, which yields high viral titers. This makes it easier and less expensive to manufacture the virus and also leads to high level expression of the therapeutic transgene payload in vivo and; (3) the insertion of a transforming growth factor beta (TGF-ß) trap transgene, which binds to and neutralizes the potently immunosuppressive cytokine, TGF-ß, that cancer cells frequently overexpress to evade immune-mediated elimination [7]. The TGF-beta trap generated by the virus inhibits TGFβ1 and TGFβ3, but not TGFβ2, from initiating the TGFβ signaling cascade in target cells.

Extensive preclinical evaluation of a murine version of AdAPT-001 in an immunocompetent murine model has demonstrated significant anti-tumor effects on injected and non-injected tumors both alone and in combination with a checkpoint inhibitor. This abscopal effect, in which non-injected lesions devoid of virus underwent growth regression is attributed to AdAPT-001-mediated activation of systemic anti-tumor immunity since CD8^+^T-cell infiltration was observed in these lesions [8].

While it is unclear exactly how this abscopal effect occurs and whether systemic anticancer activity is, in fact, mediated by global immune conditioning/stimulation, single dose biodistribution of mouse AdAPT-001 (mAdAPT-001) revealed long-term TGF-β trap persistence in the serum after intravenous and intratumoral administration, thus providing a potential explanation for the distant responses [9]. Hence, from preclinical studies, AdAPT-001 is hypothesized potentially to be a safe and active anticancer agent, which was the rationale to conduct this first-in-man Phase 1 multicenter clinical trial called BETA PRIME (ClinicalTrials.gov Identifier: NCT04673942). Based on the extremely fast replication dynamics of AdAPT-001 and the pseudoprogressive growth pattern that was observed in preclinical experiments, where tumor enlargement preceded regression, one theoretical concern prior to the start of the trial was that infiltration-inflamed tumors mimicking progressive disease would lead to an inappropriate and premature change in therapy.

### Translational relevance

AdAPT-001 is a fast-replicating oncolytic adenovirus that expresses a TGF-ß trap to overcome the immunosuppressive microenvironment endemic to refractory solid tumors. The objective of this Phase 1, open-label, single- and multiple-dose study was to determine safety and, secondarily, efficacy of AdAPT-001. The results demonstrated favorable safety and tolerability as well as efficacy with 3 confirmed partial responses and another 5 out of 19 patients (Part 2) achieved prolonged disease stabilization. In addition, a pattern of tumor flare or pseudoprogression (PsP) followed by delayed responses was observed in 20% or more of patients, which may have led in some cases to premature discontinuation from trial and an underestimation of efficacy. The observed pseudoprogression is characteristic of active immunotherapy and supports the ongoing study of combination therapy with checkpoint inhibitors augmented by AdAPT-001 to induce immunogenic intratumoral inflammation.

## Materials, patients, and methods

### AdAPT-001

AdAPT-001 has been described previously [10]. Briefly, the virus is based on a targeted 50 base pair deletion of two transcription factor-binding sites for Pea3 and one transcription factor-binding site for E2F1 in the E1a promoter region, which leads to abortive infection in normal cells and insertion of a proprietary TGF-ß Trap transgene in the anti-apoptotic E1b-19k region. The clinical batch of AdAPT-001 was manufactured under good manufacturing practices (GMP) conditions. Virus titer was adjusted to 5–6 × 10^11^ VP/mL and stored at <−60 °C with continuous temperature monitoring.

#### Study design

This was an open-label single-arm interventional phase I study using a 3 + 3 Dose-escalation safety run-in (Part 1), followed by a dose-expansion single agent (Part 2).

In part one of the study, three cohorts of three patients each were enrolled. The study was designed to define a maximal tolerated dose/recommended dose (MTD/RD) and regimen; to assess safety and tolerability; to assess immunogenicity and to assess potential anti-tumor activity of AdAPT-001 in patients with advanced solid tumors. All eligible patients had relapsed or refractory disease after standard therapy.

### Patients

Eligible patients were aged ≥18 years with superficially accessible, relapsed/refractory solid tumors and documented disease progression on or after their last regimen, as well as an Eastern Cooperative Oncology Group (ECOG) performance status of ≤2. Participants with an autoimmune disease, prior adenoviral therapy excluding AdAPT-001 or chemotherapy or immunotherapy within 14 days of initiating study drug were ineligible. Eligibility requirements also included an absolute neutrophil count ≥1.5 × 10^9^/L, a platelet count ≥75 × 10^9^/L, serum creatinine ≤1.5 × the upper limit of normal (ULN), bilirubin ≤1.5× ULN, an international normalized ratio (INR) ≤1.2, and aspartate aminotransferase/alanine aminotransferase/alkaline phosphatase ≤2.5 × the upper limit of the reference range.

Exclusion criteria included active infection, pregnant and breastfeeding patients, a history of human immunodeficiency virus (HIV), a history of hepatitis, a history of autoimmune disease or active autoimmune disease, prior adenovirotherapy excluding AdAPT-001, and chemotherapy or immunotherapy within 14 days of study treatment (although hormonal therapy including tamoxifen, aromatase inhibitors and gonadotropin releasing hormone agonists was allowed). The study was approved by local institutional review board, local institutional biologics committees, central review boards (Western Institutional Review Board (WIRB)) and central institutional biologics committees (WIRB Institutional Biologics Committee (WIRB) and Biomedical Research Association New York (BRANY IBC)). EpicentRx Inc., which developed AdAPT-001, funded the BETA PRIME clinical trial. All patients provided informed consent.

Patient details are given in Table 1 including, where available, due to the late disease stage of the patients enrolled, details of the prior therapies they had received.

**Table 1:** **A** Patient characteristics, study part 1. **B** Patient characteristics, study part 2.

A				
Patient	Age/Sex	Pathology	# Prior therapies	Baseline ECOG
01-001	65/M	Cervical Chordoma	3	1
01-002	66/M	Sacral Chordoma	6	1
01-005	70/M	Leiomyosarcoma	7	1
01-006	30/M	Sacral Chordoma	18	2
01-007	74/M	Lung Adenocarcinoma	4	1
01-008	70/F	Endometrial Adenocarcinoma	3	1
02-001	74/M	Colon Adenocarcinoma	2	1
03-001	48/F	Colonic Adenocarcinoma	4	1
03-002	79/M	Salivary Gland Adenocarcinoma	2	1
**B**				
01-010	67/M	Sacral Chordoma	14	1
01-011	59/F	Breast Invasive Ductal Carcinoma	10	2
02-003	77/M	Gastric Adenocarcinoma	8	1
02-004	33/F	Papillary Thyroid Carcinoma	0	1
02-005	64/M	Rectal Adenocarcinoma	3	1
03-003	79/M	Salivary Gland Adenocarcinoma	3	1
03-004	48/F	Uterine Leiomyosarcoma	6	0
03-001	48/F	Colonic Adenocarcinoma	4	1
03-002	79/M	Salivary Gland Adenocarcinoma	2	1
05-001	74/M	Eccrine Adenocarcinoma	0	1
05-002	82/M	Cutaneous Melanoma	5	1
06-001	43/M	Leiomyosarcoma: Sinonasal	4	0
06-002	64/M	Sacral Chordoma	8	1
06-003	52/F	Leiomyosarcoma: Retroperitoneal	12	0
06-004	75/M	Chondrosarcoma	3	2
06-005	57/F	Uterine Leiomyosarcoma	7	1
06-006	55/M	Cervical Chordoma	1	1
06-007	64/F	Mullerian Carcinosarcoma	5	1
07-001	33/M	Acinic Cell Carcinoma	7	1
07-002	57/M	Anaplastic Thyroid Carcinoma	2	1
07-003	29/F	Salivary Gland Carcinoma	1	1

All patients had progressive disease prior to enrollment.

#### Outcome measures, DLT and MTD definitions, and endpoints

The primary objective of the part 1 dose-escalation phase was to assess the safety, tolerability, maximal tolerated dose (MTD), and recommended dose of single dose, single-agent AdAPT-001. A dose-limiting toxicity (DLT) observation period of 4 weeks was established before the entry of the first patient at the next dose level. If one out of three patients experienced DLT during the treatment cycle, then six patients were to be treated at this dose level. The maximum tolerated dose (MTD) was defined as the dose level at which two of the three to six treated patients experienced DLT, and the recommended dose (RD) was the highest dose level at which no patients experienced DLT. Toxicity was classified according to the common toxicity criteria of the National Cancer Institute (NCI CTC, version 5.0). DLT was defined as any Grade 3 or higher toxicity.

The primary objective of the dose-expansion phase was to evaluate the safety and tolerability of multiple doses of AdAPT-001 monotherapy. Secondary objectives were to determine the anti-tumor activity of AdAPT-001 by objective response rates and by best overall response rates according to response evaluation criteria in Response Evaluation Criteria in Solid Tumors (RECIST) guideline (version 1.1), as well as progression-free survival and duration of response. An exploratory objective was to measure TGFβ trap concentrations in the serum.

#### Study assessments

Clinical and laboratory assessments were conducted at baseline and every 2 weeks thereafter. Baseline assessments included medical history, adverse events, concomitant medications, physical examination, complete blood count, complete metabolic profile, and CT scan. Adverse events were graded using CTCAE (Common Toxicity Criteria for Adverse Events) version 5.0. Tumor assessments were performed every 8 weeks using RECIST v. 1.1 guidelines.

#### Statistical analysis

Descriptive statistics were used for safety analyses for all patients who received one dose of AdAPT-001. Categorical and continuous data were summarized with frequencies and percentages. The efficacy population included all patients with a baseline assessment and a post-baseline tumor assessment.

### Treatment and clinical evaluation

The first, single dose, part of the study evaluated three escalating dose levels of AdAPT-001 at 2.5 × 10^11^, 5 × 10^11^, and 1 × 10^12^ vp. Patients in the second part of the study were given injections of AdAPT-001 once every 2 weeks at a dose of 1 × 10^12^ vp if the patients were clinically well enough to receive it until progression. The volume of virus injected was 1.85 mL.

Patients were assessed clinically, and computed tomography scans were also done, even though anti-tumor activity was not the primary outcome of the trial, as well as routine hematology and biochemistry, and coagulation panels. Central labs were collected on Days 1, 2, 4, 8, 15 and a adenovirus quantitative polymerase chain reaction (qPCR) was used to detect genomic viral DNA, and viral shedding via nasal and skin swabs. In addition, flow cytometry analysis of exploratory samples was conducted for immunophenotyping and cytokine expression.

### AdAPT-001 handling and injection

Virus was formulated at 5.0–6.0 × 10^11^ vp in a solution of 20 mM Tris, 25 mM NaCl, 2.5% glycerol, at pH 8, stored at ≤ −60 °C, and diluted before use when necessary.

### Virus detection

DNA was extracted from serum and VTM swabs (nasal and buccal) using quantitative PCR to detect adenovirus. Quantitative PCR was done by Eurofins Viracor.

## Results

### Patient characteristics

In Part 1, a total of 9 patients were enrolled ranging from 30 to 79 years old. A total of 8 males and 1 female were enrolled across the three cohorts as described in Table 2a.

**Table 2:** **A** Demographics and baseline characteristics by cohort and overall for part 1 of the study. **B** Demographics and baseline characteristics for part 2 of the study.

A				
Parameter	Cohort 1: 2.5 × 10^11^ VP	Cohort 2: 5.0 × 10^11^ VP	Cohort 1: 1.0 × 10^12^ VP	Total
Age (years)				
*n*	3	3	3	9
Mean (SD)	67.0 (2.6)	59.3 (25.4)	65.7 (15.9)	64.0 (15.5)
Median	66	74	70	70
Q1–Q3	65.5–68.0	52.0–74.0	59.0–74.5	65.0–74.0
min	65	30	48	30
max	70	74	79	79
QR	2.5	22	15.5	9
Missing	0	0	0	0
Sex				
Female	0 (0.0%)	0 (0.0%)	1 (33.3%)	1 (11.1%)
Male	3 (100.0%)	3 (100.0%)	2 (66.7%)	8 (88.9%)
Total	3 (100.0%)	3 (100.0%)	3 (100.0%)	9 (100.0%)
Ethnicity				
Hispanic or Latino	0 (0.0%)	1 (33.3%)	0 (0.0%)	1 (11.1%)
Non-Hispanic	3 (100.0%)	2 (66.7%)	2 (66.7%)	7 (77.8%)
Unknown	0 (0.0%)	0 (0.0%)	1 (33.3%)	1 (11.1%)
Total	3 (100.0%)	3 (100.0%)	3 (100.0%)	9 (100.0%)
Tumor type				
Chordoma	2 (66.7%)	1 (33.3%)	0 (0.0%)	3 (33.3%)
CRC	0 (0.0%)	1 (33.3%)	1 (33.3%)	2 (22.2%)
Endometrial	0 (0.0%)	0 (0.0%)	1 (33.3%)	1 (11.1%)
Leiomyosarcoma	1 (33.3%)	0 (0.0%)	0 (0.0%)	1 (11.1%)
NSCLC	0 (0.0%)	1 (33.3%)	0 (0.0%)	1 (11.1%)
Salivary	0 (0.0%)	0 (0.0%)	1 (33.3%)	1 (11.1%)
Total	3 (100.0%)	3 (100.0%)	3 (100.0%)	9 (100.0%)

B	
Parameter	Treatment: 1.0 × 10^12^ VP
Age (years)	
*n*	23
Mean (SD)	59.8 (15.4)
Median	64
Q1–Q3	50.5–73.5
min	29
max	82
IQR	23
Missing	0
Sex	
Female	9 (39.1%)
Male	14 (60.9%)
Total	23 (100.0%)
Race	
Black or African-American	3 (13.0%)
White	19 (82.6%)
Missing	1 (4.3%)
Total	23 (100.0%)

In Part 2, a total of 23 patients were enrolled ranging from 29 to 82 years old. A total of 14 males and 9 females were enrolled as described in Table 2b.

### Safety

Part 1 Dose-escalation proceeded without dose-limiting toxicities. No MTD was reached. Therefore, 1.0 × 10^12^ VP was the recommended Phase 2 dose. No treatment-related serious adverse events (SAEs) have been reported for any patient. A total of 10 non-treatment-related SAEs were reported. SAEs were not dose related. There were no complications resulting from the injection, including bleeding or infection. The most reported TEAEs were primary influenza-like symptoms such as fever, chills, arthralgia, and injection site erythema. All influenza-like illness events were grade 1 or 2, and none led to dose discontinuation. A greater number of these influenza-like symptoms were reported within 24 h of the first cycle 1 dose than after the second or third doses, and the overall frequency was higher following the first dose in cycle 1 than following the initial doses of cycle 2. The incidence of related adverse events is shown in Table 3.

**Table 3:** Incidence of related adverse events by system organ class preferred term and NCI-CTCAE grade, safety population. No grade 4 or 5 adverse events observed in part 2.

System organ class	Preferred term	Treatment	Grade 1	Grade 2	Grade 3	Total (*n* = 19)
Cardiac disorders	Tachycardia	AdAPT-001 1 × 10^12^ VP	0 (0.0)	1 (5.3)	0 (0.0)	1 (5.3)
Gastrointestinal disorders	Dry mouth	AdAPT-001 1 × 10^12^ VP	1 (5.3)	0 (0.0)	0 (0.0)	1 (5.3)
	Nausea	AdAPT-001 1 × 10^12^ VP	0 (0.0)	1 (5.3)	0 (0.0)	1 (5.3)
	Vomiting	AdAPT-001 1 × 10^12^ VP	1 (5.3)	0 (0.0)	0 (0.0)	1 (5.3)
General disorders and administration site conditions	Asthenia	AdAPT-001 1 × 10^12^ VP	1 (5.3)	0 (0.0)	0 (0.0)	1 (5.3)
	Chills	AdAPT-001 1 × 10^12^ VP	5 (26.3)	1 (5.3)	0 (0.0)	6 (31.6)
	Fatigue	AdAPT-001 1 × 10^12^ VP	1 (5.3)	1 (5.3)	0 (0.0)	2 (10.5)
	Injection related reaction	AdAPT-001 1 × 10^12^ VP	5 (26.3)	0 (0.0)	0 (0.0)	5 (26.3)
	Pyrexia	AdAPT-001 1 × 10^12^ VP	2 (10.5)	1 (5.3)	0 (0.0)	3 (15.8)
	Swelling face	AdAPT-001 1 × 10^12^ VP	1 (5.3)	0 (0.0)	0 (0.0)	1 (5.3)
Infections and infestations	Sinusitis	AdAPT-001 1 × 10^12^ VP	1 (5.3)	0 (0.0)	0 (0.0)	1 (5.3)
Injury, poisoning, and procedural complications	Injection related reaction	AdAPT-001 1 × 10^12^ VP	2 (10.5)	1 (5.3)	0 (0.0)	3 (15.8)
Investigations	Platelet count decreased	AdAPT-001 1 × 10^12^ VP	1 (5.3)	0 (0.0)	0 (0.0)	1 (5.3)
Musculoskeletal and connective tissue disorders	Arthralgia	AdAPT-001 1 × 10^12^ VP	1 (5.3)	0 (0.0)	0 (0.0)	1 (5.3)
	Neck pain	AdAPT-001 1 × 10^12^ VP	1 (5.3)	0 (0.0)	0 (0.0)	1 (5.3)
	Pain in extremity	AdAPT-001 1 × 10^12^ VP	1 (5.3)	0 (0.0)	0 (0.0)	1 (5.3)
Nervous system disorders	Dysgeusia	AdAPT-001 1 × 10^12^ VP	1 (5.3)	0 (0.0)	0 (0.0)	1 (5.3)
Skin and subcutaneous tissues disorders	Dermatitis acneiform	AdAPT-001 1 × 10^12^ VP	0 (0.0)	0 (0.0)	1 (5.3)	1 (5.3)
Vascular disorders	Hypertension	AdAPT-001 1 × 10^12^ VP	0 (0.0)	0 (0.0)	1 (5.3)	1 (5.3)

### Response

**Fig. 1:**
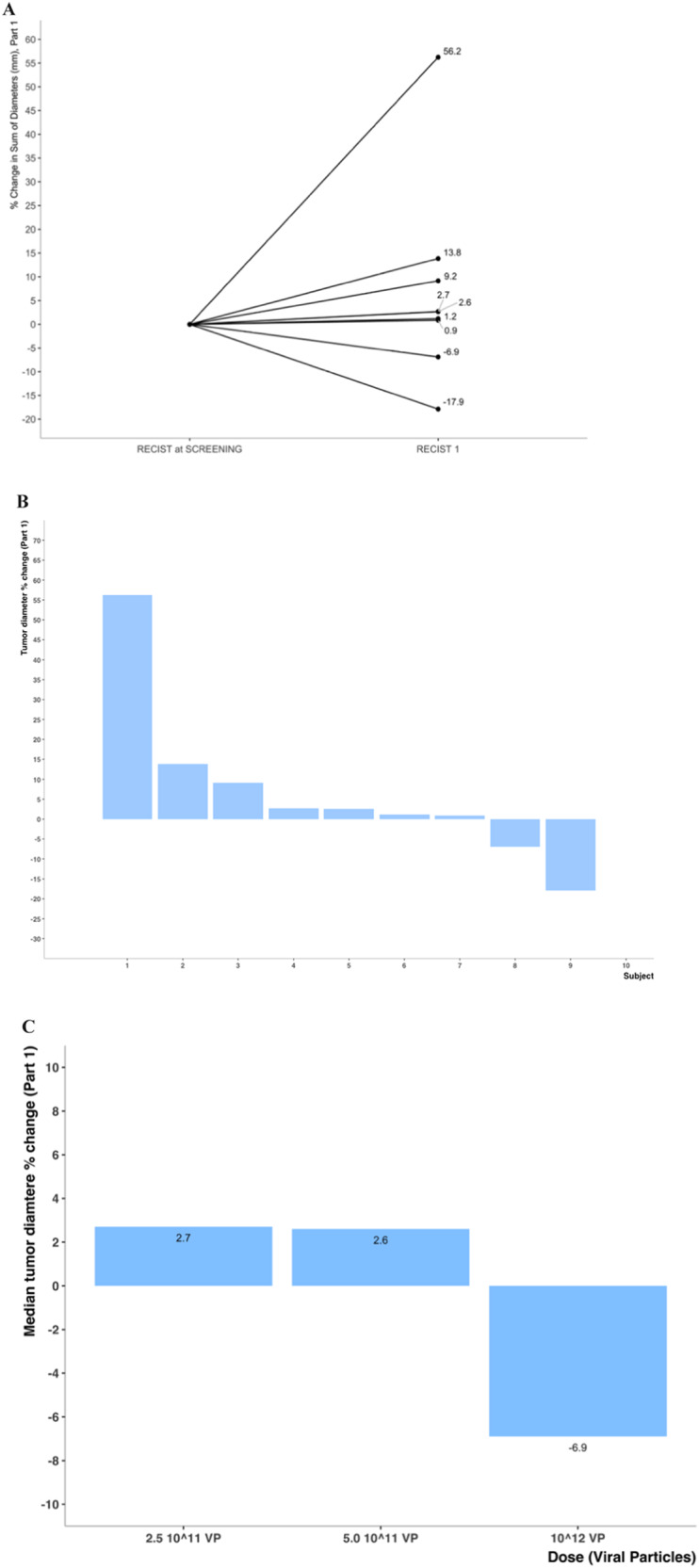
Anti-tumor activity of AdAPT-001 from Part 1 of the study. **A** Tumor diameter % change from screening, part 1 straight line graph. **B** Tumor activity descriptive statistics, part 1 bar plot. **C** Tumor response by cohort, part 1 bar plot.

In Part 1, the median number of lesions treated was 1 (range, 1–2 lesions). As this part was a single injection, patients were only followed for response at 8 weeks post-injection. Some evidence of tumor shrinkage was observed along with a possible dose response, as shown in Fig. 1 A, B, and C.

There was 1 patient (11.1%) that experienced a prolonged stable disease with just one injection, and symptomatically the patient improved as he reported resolution of chest wall pain and shortness of breath.

**Fig. 2:**
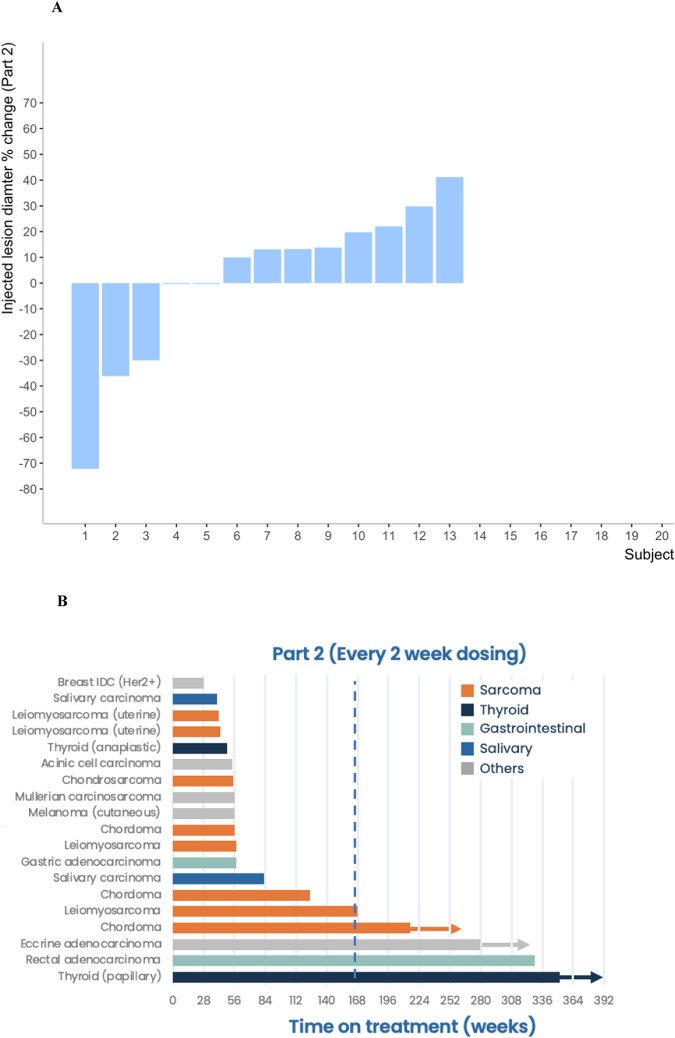
Anti-tumor activity of AdAPT-001 from Part 2 of the study. **A** Tumor diameter waterplot part 2. **B** Tumor activity swimmer’s plot with a dotted line at 6 months to indicate prolonged stability.

In Part 2, 19 of the 19 patients enrolled were evaluable. Among the 19 eligible patients, 15 were radiographically evaluable for response to therapy. Among the 15 patients evaluable for response, there were 2 (10.5%) PR (histology: thyroid and eccrine), 1 (5.3%) PR in the injected lesion (histology: chordoma), and 5 of 19 (26.3%) patients had a prolonged stable response (greater than 5.0 months; histology: thyroid, rectal, eccrine, leiomyosarcoma and chordoma). The clinical benefit rate (PR + SD) was 7 of 19 (36.8%) of patients with an average duration on trial of 6.8 months (4 patients are still on treatment as of 04/11/2023). A tumor diameter waterplot and tumor activity swimmer’s plot are shown in Fig. 2A, B.

All patients were previously treated, and all were ambulatory. In Part 2, as of April 11, 2023, patients have received a mean of 7.3 doses or 3.7 cycles (range, 2–20 doses/patient, 1–9 cycles/patient) of therapy. There are currently 4 patients still active on Part 2 of the trial. The median number of lesions treated was 2 (range, 1–5 lesions).

**Fig. 3:**
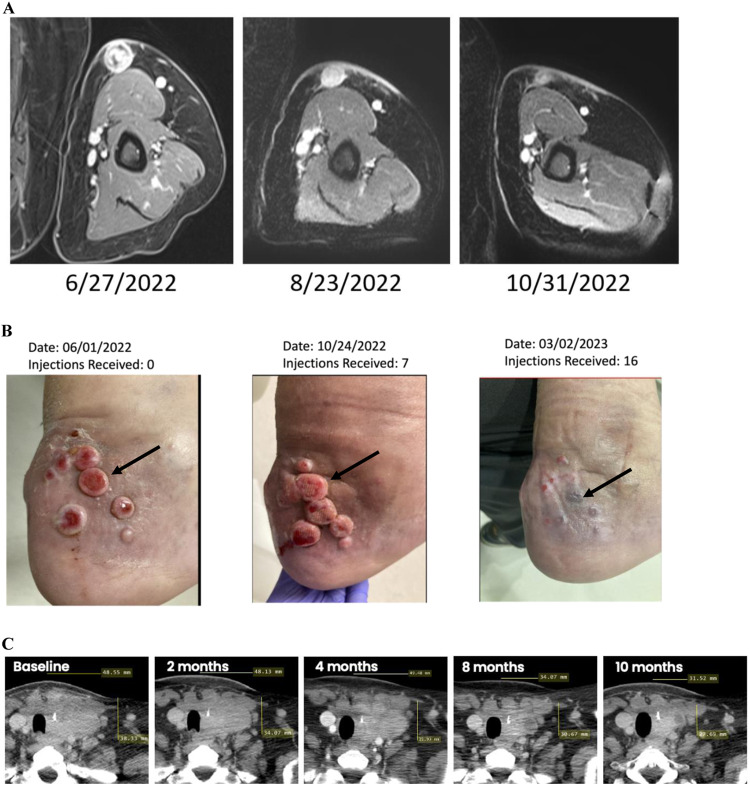
Three individual patient examples of AdAPT-001-mediated anti-tumor activity. **A** Chordoma patient treated with AdAPT-001, response in injected lesion. **B** Response of back heel lesions to AdAPT-001 over time with lesion disappearance. **C** Partial response of patient with thyroid carcinoma charted over time.

Three examples of favorable treatment responses are described below in Fig. 3A, B and C. One of the responses only occurred after initial tumor enlargement, indicative of pseudoprogression.

The first is patient 006-002, a 64-year-old male patient with recurrent sacral chordoma metastatic to the arm, liver, kidneys, and retroperitoneum that received radiotherapy to the sacrum. This was followed 7 months later by Afatinib and DEB-TACE. The patient enrolled on BETA PRIME in June 2022 and received 8 injections over a four-month period. Although *a 72.2% reduction in his injected lesion in the arm has been observed (see MRI images above)*, he discontinued treatment in November 2022 due to RECIST-defined progression at non-injected metastases. However, the fact that the injected tumor continues to have an ongoing response strongly suggests a treatment-related effect. The patient remained on study for 113 days.

The second is a 75-year-old male patient with metastatic eccrine carcinoma whose initial presentation included 6 lesions on the inside ankle/back heel, one open ulcerated lesion on the left outside ankle/lower leg, and one ulcerated lesion in his left hamstring area. These lesions were accompanied by significant left lower limb lymphedema, which led to falls, reduced mobility, social isolation, and the need for mobility aides. The patient was injected in the lesion 3 as noted below by the arrow for all injections to date. After approximately 4 months on treatment (7 injections of AdAPT-001), the patient experienced a temporary inflammatory reaction, resembling a tumor flare, also known as pseudoprogression (Fig. 3). The lesions on his heel, outside ankle, and hamstring were visibly enlarged at 4 months, however because the patient reported symptomatic improvement in pain, mobility, and swelling, the decision was made to continue treatment. At month 5 lesions began to shrink, and led to what is currently a durable partial response.

#### Correlative data

**Fig. 4:**
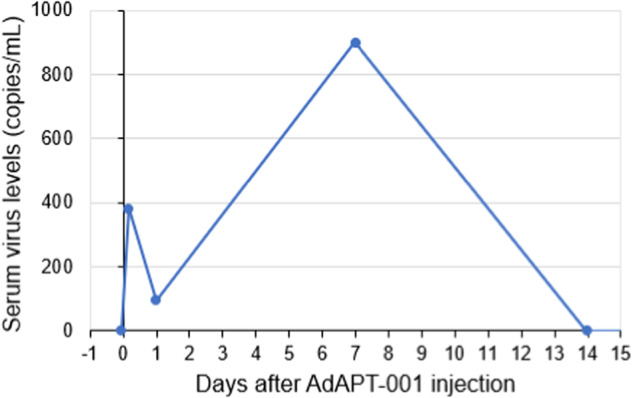
Serum adenovirus levels in patient 001-006. Serum levels fell to below the limit of quantification 1 day after injection (less than 190 copies/mL, plotted as 95) and increased at 7 days after injection.

Serum samples were analyzed for circulating adenoviral DNA during cycle 1, before patients would be expected to develop or boost anti-adenovirus antibodies in response to treatment with AdAPT-001. Most samples collected during the four-hour post-injection observation period of the first dose had no or below the limit of quantification copy numbers, consistent with AdAPT-001 remaining within the injected tumors. Five patients had detectable viral genomes in the serum on Day 4 or Day 8 at levels higher than the preceding timepoint (See Fig. 4), suggesting that AdAPT-001 replication occurred in the tumors with new virions being shed into the circulation. While this serves as evidence of viral replication, the hypothesized mechanism of action of AdAPT-001 is induction of an immune response against the cancer with systemic activity and is not dependent on virus circulating to infect distant tumors. At the time of the paper, 10 patients (Part 1 and Part 2) had detectable adenoviral DNA on injection site swabs up to Day 22. No significant shedding was detected in nasopharyngeal swabs.

## Discussion

AdAPT-001 administration in this Phase 1 first-in-man trial demonstrated acceptable safety and tolerability with no treatment-related grade 4 adverse effects, no dose-limiting toxicities and no MTD was reached. In terms of efficacy, out of 15 radiographically evaluable patients, 3 had a partial response, and 5 had prolonged stable disease ≥6 months.

AdAPT-001, like ONYX-015, the first oncolytic adenovirus (OAV) to enter clinical trials in 1996, is an attenuated, tumor-selective adenovirus [11]. However, AdAPT-001 is attenuated by deletion of a domain of the E1a enhancer/promotor region and, unlike ONYX-015, retains the E1b-55k gene. AdAPT-001 replicates many folds faster than ONYX-015, or any of the several gene-attenuated and/or pseudotyped i.e., fiber substituted ONYX-015 derivatives that have entered clinical trials or that are in development. This is because the AdAPT-001 Ad5 base oncolytic vector is not targeted to tumors through capsid modification, the insertion of exogenous cancer-specific promoters or hybridization of adenoviral serotypes, strategies that are currently in clinical use to modify the natural tropism of adenoviruses. Instead, AdAPT-001 is detargeted from non-tumor cells through the deletion of a small 50 base pair region located upstream of the E1A initiation site, which leads to abortive infection and no or restricted cytolytic activity in normal cells, but potent near wild type levels of replication, expression, and cytolytic activity in tumor cells [10–12].

Such minimal modification differentiates AdAPT-001 from the slowed-down, attenuated kinetics of other adenoviruses that are, for example, pseudotyped with capsids from different Ad serotypes, that are E3 gene-deleted, and that are chimerized with exogenous DNA promoters, all potentially to the detriment of transfection efficiency, protein expression, and manufacturability [13, 14]. AdAPT-001 is manufactured to cGMP standards “in house”.

The other modification in AdAPT-001 is deletion of the E1B-19K gene—a Bcl-2 adenoviral homolog that potently inhibits apoptosis [15]—and its replacement with a Transforming Growth Factor-beta (TGF-β) ligand “trap”; this trap is a TGFβ receptor ectodomain-IgG Fc fusion protein, which binds to and neutralizes the potently immunosuppressive and fibrosis-inducing cytokine, TGF-β [9].

Tumors with an immune-devoid phenotype are known as “cold” and these are generally unresponsive to checkpoint inhibitors. Of interest, then, is the combination of AdAPT-001 with checkpoint inhibitors, as the combination of viral infection and the expression of the TGF-β trap transgene may induce lymphocytic infiltration and prime for checkpoint inhibition efficacy since high levels of the TGF-β cytokine are associated with poorly T-cell infiltrated, “cold” tumors, which are resistant to immunotherapy [15]. That said, cancer is a systemic disease and for a localized therapy like AdAPT-001 to systemically treat and control cancer as a single agent, especially in the setting of gross metastatic disease, requires the induction of an abscopal effect in which tumor-reactive T cells within the injected tumor migrate to and eliminate noninjected or “secondary” tumors [16].

In this Phase 1 trial evidence of a distant, potentially immune-mediated effects were observed as two cases of uninjected areas regressed and up to five cases, and possibly more, underwent apparent pseudoprogression, defined as an initial increase of the volume, significant clinical improvement, and/or number of tumors compared to baseline. This made it initially difficult to determine whether true progression occurred and whether discontinuation of therapy was warranted, prior to an observed decrease below baseline values over two months later that indicated a favorable treatment response. Such a high rate of apparent pseudoprogression strongly suggests the need for adjuncts or aids including biopsy, measurement of cell-free DNA, PET imaging and ultrasound as well as a high index of clinical suspicion to help differentiate between pseudoprogression and true progression so that treatment, either as monotherapy or in combination with checkpoint inhibitors, is not inappropriately stopped (or continued).

This study would have undoubtedly benefited from pathologic evaluation of tumors to demonstrate the replication and distribution of the oncolytic virus, tumor shrinkage, necrosis of the cancer cells, and immune infiltration and to confirm pseudoprogression. However, because no patients consented to the optional biopsies, no pathological results are available.

One of the reviewers of this manuscript suggested to correlate TGF-β levels with patients’ outcomes. Unfortunately, however, it is not possible to characterize intratumoral immunologic responses to treatment and intratumoral TGF-β trap expression because on-treatment biopsies were not mandated. While other TGF-β inhibitors have been limited by side effects when given systemically, intratumoral expression of the TGF-β trap by AdAPT-001 leads to high intratumoral and lower systemic levels of the trap. Therefore, measurements of TGF-β levels would require on-treatment biopsies, potentially at multiple timepoints due to the dynamic nature of an immune response to a viral agent. That said, the response patterns including delayed response and pseudoprogression before response are highly suggestive of an immunologic mechanism of action.

In conclusion, AdAPT-001 is well tolerated at 1.0 × 10^12^ vp given every two weeks, which is the dosing schedule to be taken forward in further studies. Biological activity was observed, even at the lowest dose of 2.5 × 10^11^ vp, as evidenced by local reactions, tumor flattening, pseudoprogression, and virus replication. The promising results from this trial provide further impetus to evaluate AdAPT-001 in several phase 2 clinical trials in individual tumor types with or without checkpoint inhibitors.

## Data Availability

The datasets generated during and/or analyzed during the current study are available from the corresponding author on reasonable request. The authors confirm that the data supporting the findings of this study are available within the article.
